# Insufficiently studied factors related to burnout in nursing: Results from an e-Delphi study

**DOI:** 10.1371/journal.pone.0175352

**Published:** 2017-04-07

**Authors:** Guadalupe Manzano-García, Juan-Carlos Ayala

**Affiliations:** 1 Department of Sciences Education, University of La Rioja, Logroño, La Rioja, Spain; 2 Department of Economics and Business, University of La Rioja, Logroño, La Rioja, Spain; Waseda University, JAPAN

## Abstract

**Objective:**

This study aimed to identify potentially important factors in explaining burnout in nursing that have been insufficiently studied or ignored.

**Methods:**

A three-round Delphi study via e-mail correspondence was conducted, with a group of 40 European experts. The e-Delphi questionnaire consisted of 52 factors identified from a literature review. Experts rated and scored the importance of factors in the occurrence of burnout and the degree of attention given by researchers to each of the variables listed, on a six-point Likert scale. We used the agreement percentage (>80%) to measure the level of consensus between experts. Furthermore, to confirm the level of consensus, we also calculated mean scores and modes. Regardless of the degree of consensus reached by the experts, we have calculated the mean of the stability of the answers for each expert (individual's qualitative stability) and the mean of the stability percentages of the experts (qualitative group stability).

**Results:**

The response rate in the three rounds was 93.02% (n = 40). Eight new factors were suggested in the first round. After modified, the e-Delphi questionnaire in the second and third rounds had 60 factors. All the factors reached the third round with a consensus level above 80% in terms of the attention that researchers gave them in their studies. Moreover, the data show a total mean qualitative group stability of 96.21%. In the third round 9 factors were classified by experts as ‘studied very little’, 17 as ‘studied little’ and 34 as 'well studied'

**Conclusion:**

Findings show that not all the factors that may influence nursing burnout have received the same attention from researchers. The panel of experts has identified factors that, although important in explaining burnout, have been poorly studied or even forgotten. Our results suggest that further study into factors such as a lack of recognition of part of the tasks that nurses perform, feminine stereotype or excessive bureaucracy is needed for a better understanding of this syndrome and improve the quality of life in nurses.

## Introduction

Burnout is the result of an internal conflict that opposes or hinders the employment of personal values in projects of the company [1]. Nursing staff have certain professional expectations and inclinations that make them susceptible to suffering burnout. Working conditions of nurses (work overload, excessively long working days, role ambiguity and conflict, lack of autonomy…) that are increasingly difficult to cope with result in a progressive loss of idealism and energy, the reasons that led to the choice of this profession [2]. Before long, the nurse starts to experience emotional exhaustion, depersonalisation, and reduced personal fulfilment. These are the three main dimensions of burnout syndrome measured by the Maslach Burnout Inventory (MBI) [3].

Emotional exhaustion is considered the central factor of burnout syndrome [4, 5]. It is characterised by a feeling of exhaustion, lack of energy and physical and emotional overload. The individual feels exhausted and unmotivated, being unable to relax. The phenomenon of depersonalisation results in negative attitudes and feelings in addition to insensitivity at work towards patients. It represents the interpersonal context in burnout. This state describes depersonalisation; the individual belittles their relationship with colleagues, patients and the organisation they work for. It is characterised by an emotional numbing and concealment of emotions; its most common symptoms are anxiety, increased irritability, lack of motivation, a reduction in idealism, despair, selfishness and alienation. The manifestations of depersonalisation reflect the individual's attempts to adapt to the situation and ease tensions by reducing contact with people [6].

A lack of personal fulfilment refers to a feeling of incompetence and lack of satisfaction and productivity at work. An example that explains this state well is the moment the professional begins to question their choice of profession, calling into question their ability to carry it out. Work no longer fulfils the individual, they feel inadequate both personally and professionally; this behaviour affects their ability to carry out their work and the contact they have with people, drastically reducing their productivity [7] and the quality of the services provided to patients [8].

The MBI is an international benchmark to measure burnout; the internal consistency of its three scales has been confirmed in numerous studies. In a review of the literature on factorial structure and the psychometric property of MBI covering the years 2000 to 2014, Loera, Converso and Viotti [9] retrieved 12 works which confirm the structure of three factors. Amongst them, there are 7 works (58.3%) in which the analysed sample covers nurses alone. The recurrence of very strong and highly statistically significant findings using MBI has been often interpreted as a confirmation for the validity of the concept. The use of MBI has been so ample that burnout has become what the MBI measures. This tautology is conditioning research on burnout [10] since the concept of burnout exceeds what the MBI measures. On the other hand, many studies about burnout have been conducted adding in one or two variables that had not been previously examined (but have mostly explicitly replicated earlier findings). However, the relationship of burnout to other obviously related psychological processes and concepts (such as depression) has not been sufficiently clarified [11], nor have cultural factors in the definition and experience of burnout ever been fully explored. Perhaps what is needed is a critical approach that furthers the conceptual basis of burnout and its lines of development [12].

There are different but complementary psychological explanations for burnout. These explanations focus on the importance of the individual, interpersonal relations and organisational and social factors [13]. Burnout affects a huge amount of people working in a wide variety of professions (firefighters, teachers, prison guards, drivers, etc.) [14]. Amongst the professions which have been described by their high-risk to suffer from burnout are those related with health care (nurses, doctors, pharmacists, etc.) [15] as a result of experiencing high levels of emotional strain, owing to stressful working environments exacerbated by sick and dying patients to whom they provide care [16–17]. Burnout has been studied extensively in nursing. Nursing has been found to be one of the professions experiencing high levels of burnout [18]. These results may be explained, for example, in the light of the effort-reward model. During the interaction between the individual and her working environment, demands exceed the individual resources and the nurse experiences an effort-reward imbalance. In the long run, the stressful experiences resulting from this disequilibrium are very likely to result in states of exhaustion and burnout [19]. Due to the nature of their work [12], in nurses, this disequilibrium increases by reason of the role conflict, the role's ambiguity, the bad relationships at work, the lack of staff, overtime hours, inadequate salaries, lack of promotion, scarce hierarchical support, etc. [20, 17].

The prevalence of burnout syndrome in nurses is variable and is related to their working shift. For example, Albendín et al. [21] carried out a revision of 27 works undertaken in 13 different countries (Spain, Brazil, United Kingdom, United States, Chile, China, Egypt, Greece, Holland, Iran, Ireland, Mexico and Turkey) and found a high burnout prevalence rate of 32.2% in nurses working in emergency services. In France, Poncet et al. [22], on a sample of 2,392 nurses who worked in 165 intensive care units, found out that 28.4% showed severe burnout degrees and 60% showed high degrees in at least one of its dimensions. In Spain, San Clemente et al. [23] evinced that burnout figures in nursing professionals range between 18% and 33%. Between 26.74% and 55% Spanish nursing professionals show emotional exhaustion. In Belgium, Vander et al [24], using a sample of 675 health care nursing homes found a 12.7% level of prevalence. In the United States, Aiken et al. [25], on a sample of 10,000 nurses at hospitals in Pennsylvania concluded that 43% were emotionally exhausted. In Brazil, das Merces et al [26], on a sample of 189 Primary Health Care nursing practitioners, found that the prevalence of burnout syndrome was 10.6%. However, these results should be viewed with caution [27]. On one hand, the cut-off points for differentiating between burnout “cases” and “non-cases” are based on arbitrary statistical norms. On the other hand, studies have often been conducted on relatively small and non-representative samples, using different measurement instruments or being conducted in culturally different countries [27].

On a daily basis, nursing staff suffer a variety of stressors that have been recognised and studied in previous papers as antecedents to burnout syndrome [14]. Stressors affecting nurses are related to their professional performance (e.g. autonomy) [28]. Stressors related to their work environment (e.g. managerial support for nursing, doctor-nurse collegial relations, promotion of care quality) [29], social support (e.g. friends, family relatives, colleagues, supervisors) [30,31], the type of assistance they provide (e.g. primary care, tertiary hospital, rural) [32], their lifestyle (e.g. physical exercise, sleeping hours, diet) [33], personality traits (e.g. type A personality, extraversion) [34] or the economic and social context [35] have also been described and studied.

Several studies have conducted systematic reviews about factors contributing to burnout in nurses [17]. They have been classified into different groups or families. However, we do not know of any previous studies that have hierarchically categorised the relative importance of these factors in the occurrence of the syndrome or which have attempted to identify factors not yet studied. Not all factors considered antecedents to burnout in nurses have the same importance, nor have they been explored to the same extent by researchers. One of the reasons for this gap may be partially due to the difficulty of objectively measuring stressors, which accentuate the occurrence of the syndrome within this group (e.g., constant interruptions while working) and, another, to the difficulty of securing nurses' commitment and collaboration to measure these variables. Thus, the overall purpose of this study was to establish a list of important factors in the development of burnout in nurses and the level of attention given to each of those factors by previous research studies. The specific objectives were: (1) create a list of the factors, that is as complete as possible, influencing burnout using published studies which have focused exclusively on samples of nurses; (2) create a list of potentially important factors that have been ignored when explaining burnout; (3) prioritise and reach a consensus on the attention paid by researchers to factors that explain burnout.

In general, our study contributes to research on burnout in two ways. First, we extend the existing literature identifying factors which, despite being important for a better understanding of the syndrome, have not been explored sufficiently by researchers. Second, this work contributes to identify potential future research on burnout in nurses. As a result, this research will allow for a better understanding of the explanatory factors of burnout, which will contribute to a more comprehensive understanding of the syndrome.

## Methodology

### Ethics statement

The work follows the rules set by the Academic Commission of the Faculty of Business Sciences of the University of La Rioja, to which the signers of the work belong. All procedures followed were in accordance with the ethical standards of the responsible committee on human experimentation (institutional and national) and with the Helsinki Declaration of 1975, as revised in 2000. The Commission reviewed and approved the ethical guidelines of research before the study began. Informed consent was obtained from all patients for being included in the study. All experts worked anonymously and independently of each other.

### Design

The first step in the research study was to conduct computerised keyword searches in the Web of Knowledge, Cinahl Plus, Medline and Journal Citation Reports databases. The search was based on the methods described by the Centre for Reviews and Dissemination [36]. The number of investigations on burnout has increased significantly during the first fifteen years of the 21st century. From the years 2000 to 2014, both included, the consulted databases have shown a total of 5,351 references to scientific articles and books on burnout, from which 636 (12%) were carried out with nurses. From the years 1900 to 2000 showed 2,273 references, out of which 242 (10.5%) were carried out with nurses. This increased attention for the syndrome during the last 15 years is due to its consequences which are harmful to both the individual and the organisation. [27]. Therefore, in order to generate a list of all possible factors related with burnout in nurses which have been researched during the last 15 years, we reviewed peer-reviewed journal articles published from 2000 onwards (between January 2000 and July 2014) where ‘burnout’ appeared in the title and ‘nursing’ in the topic. Other inclusion criteria for the articles were that they: a) be published in the English language; b) be an empirical study; c) identify and describe the factors of burnout that have been studied and d) exclusively use samples of European nurses. The articles were screened by the first author (GM) to eliminate duplicated studies and those not meeting the inclusion criteria. The resulting number of articles complying with the inclusion criteria amounted to 54, from which 4 (7.4%) were longitudinal. Next, both authors (GM and JCA) analysed the articles separately to extract the factors associated with burnout. They then reviewed the extracted data to assess consensus and ensure all factors had been identified. Finally we classified the factors of burnout based on a taxonomy conducted by Maslach, Schaufeli and Leiter [37] or Maslach and Leiter [38] who propose that factors related with burnout may be classified in two big groups: situational factors (job characteristics, occupational characteristics and organizational characteristics) and individual factors (demographic variables and personality characteristics). Disagreements were resolved by discussion.

Basing ourselves on the list of factors obtained, the next step was to identify, according to the experts, whether these factors are important for the occurrence of burnout in nurses and the degree to which they have been studied by researchers. For this, we used the e-Delphi technique, a social research technique whose purpose is to obtain a reliable group opinion from a set of experts [39]. This technique transforms experts' individual evaluations into collective judgements during the research study. It is a structured process that collects and condenses knowledge from a group of experts through a series of questionnaires interspersed with controlled opinion feedback. The response-analysis-feedback-response process was repeated 3 times, until experts reached a consensus level of 80%.

### Participants

Triandis et al. [40] showed that in different cultures, the same concept can have different meanings. For Moreno et al. [12] this occurs with burnout syndrome. Moreover, Berry et al. [41] have shown that cultural factors are relevant in the explanation of psychological processes. From this perspective, socio-cultural values may affect the importance given to the factors involved in the onset of the syndrome [42, 43] and how they are interpreted. For this reason, it would be interesting to study whether experts from different countries agree with the level of importance given to the factors that researchers have linked to burnout.

Using contacts with colleague nurses and leaders from different European countries, we compiled a sample of potential burnout experts who met our inclusion criteria. The panel of experts was made up of 43 nursing teachers who worked in different nursing schools in Spain (12 nursing teachers), Portugal (8 nursing teachers), France (7 nursing teachers), Belgium (6 nursing teachers), England (5 nursing teachers) and Romania (5 nursing teachers). All our panel experts had five characteristics in common, they: a) were a registered nurse; b) had a degree or a post-graduate degree in nursing; c) had published at least two papers on burnout in the last 5 years in impact international journals (Journal Citation Report–JCR-); d) in addition to being teachers, they work in the nursing profession; (e) had more than 15 years' experience in the profession. This criteria for selecting experts ensured that all participants had prior training and experience and were considered suitable for making accurate judgements. The experts worked anonymously and independently of each other.

The first round of questionnaires were sent to 43 experts. Three of them decided not to participate in the study and provided no feedback. The 40 experts who participated in round I also participated in rounds II and III. This level of participation was probably due to the previous contacts made with experts through their colleagues or leaders.

### Data collection

All the questionnaires were sent to the experts by e-mail. The first round took place in the first half of October and the two subsequent rounds took place in November 2014. To maximise the return rate, a phone call was made 2 days after the mailing in all cases with a follow-up email 1 week later

The first e-Delphi round was a qualitative round. Each expert received the list of factors identified in the review of the scientific studies. Using a binary Likert scale where 0 = No and 1 = Yes, the experts were asked to rate each factor according to whether or not they considered it important in explaining burnout in nurses. Experts were also asked an open question: “Considering the list of factors that you have just evaluated, please add any factors that are not listed and which are important in the occurrence of burnout in nurses.” Each expert also received an explanation of the e-Delphi objectives in addition to the practical conditions of the questionnaire (response time and anonymity guarantee).

In the second round the experts were presented with the variables resulting from the previous round and were asked to rate their importance in the occurrence of burnout and the degree of attention given by researchers to each of the variables listed, on a Liker scale from 1 ‘important and well-studied’ to 6 ‘very important and studied very little’ (scores grouped 1 and 2 = well studied; 3 and 4 = studied little; or 5 and 6 = studied very little).

In the third round experts were asked to repeat the assessment of each burnout factor taking into account the scores given by their anonymous colleagues in the previous round. In this third round each expert received: a) the questionnaire; b) the assessment made of each factor in the previous round and; c) the results of the statistical analysis taken from the group responses for each factor in the previous round: mean, mode, minimum, maximum and standard deviation. This procedure is an essential part of the e-Delphi method, and results in consensus and stability in the answers [44].

### Data analysis

We used IBM SPSS 22.0 for the quantitative analysis. In the second and third e-Delphi round descriptive statistics were generated and frequencies, percentages and cumulative percentages were used to determine the degree of agreement among experts. Typically, rates of agreement or consensus between 70 and 80% were acceptable [45]. We used the agreement percentage to measure the level of consensus between experts. A variable is included in the ‘studied very little’ group if at least 80% of participants gave it a score of 5 or 6. A variable is included in the ‘studied little’ group when at least 80% of participants gave it a score of 3 or 4; and is included in the 'well studied' group when at least 80% of participants gave it a score of 1 or 2. Furthermore, to confirm the level of consensus, we also calculated mean scores and modes [46].

The purpose of the successive consultations was to decrease the degree of dispersion or increase the degree of consensus in the experts' answers. In the third round each expert expressed their opinion after having observed and reflected on the opinions of other experts (the supplementary information provided about central tendency and dispersion). Typically, the distribution of responses for each round exhibited lower dispersion (or greater consensus) than that found in the previous round. To measure the degree of dispersion we used the standard deviation of the different distributions and from this point the mean score from rounds II and III. The difference between the standard deviation between the two rounds gives an idea of the variation in the degree of consensus.

Regardless of the degree of consensus reached by the experts, some authors believe that the rounds should end when the stability of the answers from the experts is very high [47]. In other words, the rounds of consultations must end if each expert, having observed and learned of the group's opinion, does not change their opinions. The stability of the answers was calculated for each expert by obtaining the percentage of factors for which their assessment in the second and third round coincided (individual's qualitative stability). Based on this measure, we have calculated the mean of the stability percentages of the experts (qualitative group stability).

## Results

The experts' age range was between 40 and 53, although most of them (82.5%) were aged between 41 and 50 (Table 1). The mean age was 46.5 years (SD = 3.43). The mean years of professional experience for each expert was 24.68 (SD = 3.71) for nursing and 18.75 (SD = 3.19) for nursing teacher. On average they have published 3.85 articles about burnout during the last 5 years (SD = 1.25; range between 2 and 7).

**Table 1:** Demographic details of the sample of experts.

	Number	%
Age (Years)		
35–40	2	5.00
41–45	13	32.50
46–50	20	50.00
51–55	5	12.50
Time working as a nurse		
15–20	5	12.50
21–25	20	50.00
26–30	11	27.50
31–35	4	10.00
Time working as a teacher		
10–15	7	17.50
16–20	19	47.50
21–25	13	32.50
26–30	1	2.50
Articles published about Burnout during the last five years		
2	3	7.50
3	16	40.00
4	12	30.00
5	4	10.00
6	3	7.50
7	2	5.00

Our research into scientific studies reporting factors that lead to the onset of burnout in nurses provided 66 different articles of which we eliminated six because they were repeated and a further six because they included doctors in their samples. The next step in the analysis was to review the 54 articles and compile the variables that could contribute to the onset of burnout. We found 52 different factors (Table 2), which were grouped into four large blocks: socio-demographic characteristics (9.62% of the factors); personality traits (23.08% of the factors); lifestyle (3.85% of the factors) and organisational factors and work environment (63.45% of the factors).

**Table 2:** Topics researched.

**INDIVIDUAL FACTORS**
**Socio-demographic characteristics**
• Age
• Gender
• Marital status
• Qualification level
• Years of working experience
**Personality characteristics**
• Personality type A
• Problem-solving skills
• Excessive enthusiasm for work with high personal ambition
• Psychological imbalance
• Empathy and compassion
• Need for social support
• Perfectionist tendencies
• Pessimism
• Poor integration at work
• Lack of belief in what is being done
• Low self-esteem
• Unable to make sense of the work being done
**Lifestyle**
• Does not perform leisure or recreation activities
• Does not sleep sufficient hours
• No free time
**SITUATIONAL FACTORS**
**Organisational factors and work environment**
• Too much work and little rest
• Organisational environment
• Lack of clarity in processes and procedures
• Lack of clarity regarding the organisation's mission and philosophy
• Few opportunities for promotion and growth
• Unclear requirements
• Impossible demands
• Patients who are vulnerable and need attention
• Emotional requirements to respond according to patient complaints and everity
• Inadequate preparation for certain services
• Job overload
• Conflicts of responsibilities
• Ambiguity in the role being performed
• Information and control
• Time given to carry out tasks
• Afraid to make mistakes and be reported
• Lack of personal control over the environment and daily decisions
• Lack of recognition and praise for a job well done
• Poor communication and unclear job expectations
• Insufficient financial compensation for the work performed
• Poor leadership
• Mental overload due to the continuous concentration that certain patients require
• Pressure of time-limits
• Monotonous work
• Lack of autonomy in decision-making
• Work complexity
• Night shifts
• Doctor and nurse conflicts
• Negative results, treatment and uncertainty in patients
• High physical demands
• Poor relationships with colleagues and superiors
• Continuously adapting to new technological procedures

In the first e-Delphi round all experts agreed that the 52 factors identified in the first stage of the research study as important for the explanation of burnout experienced by nurses. They added an additional eight factors: feminine stereotype, continuous and excessive interruptions, excessive bureaucracy, illness suffered by the subjects or their children in the past 12 months, economic difficulties, non-equitable workload (depending on the services), a lack of recognition of part of the tasks that nurses perform and mental concentration and alertness.

All the factors listed in the e-Delphi questionnaire reached the third round with a consensus level above 80% in terms of the attention that researchers gave them in their studies. From round II to III, 47 factors (78.33%) improved their level of consensus and 52 variables (86.67%) decreased their standard deviation. None of the factors' level of consensus decreased nor did their standard deviation increase between rounds II and III. In Round II the mean value of the standard deviations in the probability distribution for the 60 factors considered was 0.93. In Round III this variable reached 0.82. That is, between rounds II and III an increase in the degree of consensus is seen. Moreover, the data show a total mean qualitative group stability of 96.21%. This means that individual answers per variable only differ approximately 4% from round II to III.

The hierarchy of factors according to the degree to which they have been studied by researchers was initially conducted using the mean. When two factors had the same mean, the one with the lowest standard deviation was classified first. When two factors had the same mean and standard deviation, the one that showed a greater consensus percentage was classified first.

Table 3 shows the factors classified as ‘studied very little’ (nine factors) after three e-Delphi rounds. The table shows the classification of each factor in rounds II and III as well as the mean, standard deviation and consensus percentage for both rounds. It is worth noting that the two factors classified at the top of ‘studied very little’–‘lack of recognition of part of the tasks that nurses perform (invisible care)’ and ‘feminine stereotype’—were both top for rounds II and III. In both rounds these two factors achieved the same level of consensus, the same standard deviation and identical mean.

**Table 3:** Factors classified as ‘studied very little’. Ranking, means and consensus in rounds II and III.

Factor	Round III				Round II			
	Ranking	Mean	SD	Consensus	Ranking	Mean	SD	Consensus
The lack of recognition of part of the tasks that nurses perform (invisible care)	1	5.25	0.87	82.50%	1	5.25	0.87	82.50%
The feminine stereotype	2	5.20	0.94	80.00%	2	5.20	0.94	80.00%
Continuous and excessive interruptions	3	5.15	0.70	82.50%	8	4.93	1.00	70.00%
The economic difficulties	4	5.15	0.77	82.50%	4	5.05	0.96	80.00%
Continuously adapting to new technological procedures	5	5.15	0.92	80.00%	3	5.00	1.15	75.00%
Excessive bureaucracy	6	5.15	0.92	80.00%	6	5.15	0.92	80.00%
Illness suffered by the subjects or their children in the past 12 months	7	5.10	0.81	82.50%	5	5.03	0.95	80.00%
Non-equitable work load (depending on the services);	8	5.05	0.75	85.00%	9	4.80	1.07	72.50%
Mental concentration and alertness	9	5.05	0.75	85.00%	7	4.93	1.00	82.50%

There were 17 factors classified as ‘studied little’ (Table 4). All were classified as ‘studied little’ in both rounds II and III. The two top factors in this group were 'lack of recognition and praise for a job well done' and 'empathy and compassion'. Both factors increased in their level of consensus in round III.

**Table 4:** Factors classified as ‘studied little’. Ranking, means and consensus in rounds II and III.

Factor	Round III				Round II			
	Ranking	Mean	SD	Consensus	Ranking	Mean	SD	Consensus
Lack of recognition and praise for a job well done	10	3.38	0.81	85.00%	10	3.40	0.84	82.50%
Empathy and compassion	11	3.23	0.83	90.00%	11	3.25	0.87	87.50%
Impossible demands	12	3.20	0.88	80.00%	13	3.20	0.88	80.00%
Excessive enthusiasm for work with high personal ambition	13	3.15	0.83	87.50%	16	3.18	0.87	85.00%
Problem-solving skills	14	3.15	0.86	85.00%	17	3.18	0.90	82.50%
Perfectionist tendencies	15	3.13	0.72	85.00%	25	3.03	0.92	75.00%
Psychological imbalance	16	3.13	0.79	90.00%	15	3.18	0.84	87.50%
Pessimism	17	3.13	0.88	82.50%	14	3.20	0.97	77.50%
Low self-esteem	18	3.10	0.78	90.00%	18	3.13	0.82	87.50%
Poor integration at work	19	3.10	0.81	82.50%	19	3.10	0.81	82.50%
Lack of belief in what is being done	20	3.10	0.90	85.00%	21	3.08	0.92	82.50%
Unable to make sense of the work being done	21	3.10	0.93	82.50%	20	3.10	0.93	82.50%
Too much work and little rest	22	3.05	0.68	85.00%	12	3.20	0.85	77.50%
Lack of clarity regarding the organisation's mission and philosophy	23	3.03	0.70	92.50%	22	3.03	0.73	90.00%
Lack of clarity in processes and procedures	24	3.03	0.89	82.50%	23	3.03	0.89	82.50%
Patients who are vulnerable and need attention	25	3.00	0.85	85.00%	24	3.03	0.89	82.50%
Emotional requirements to respond according to patient complaints and severity	26	2.93	0.86	80.00%	26	2.85	0.92	75.00%

There were 34 factors classified by experts as 'well studied' (Table 5). The two factors that showed a lower mean in rounds II and III were sex and age, also being two factors that appear in most of the research on burnout.

**Table 5:** Factors classified as 'well studied'. Ranking, means and consensus in rounds II and III.

Factor	Round III				Round II			
	Ranking	Mean	SD	Consensus	Ranking	Mean	SD	Consensus
Gender	27	2.00	0.78	80.00%	29	2.08	0.92	77.50%
Age	28	2.00	0.91	85.00%	31	2.03	0.97	85.00%
Qualification level	29	1.95	0.81	80.00%	27	2.18	1.08	70.00%
Years of work experience	30	1.95	0.85	82.50%	33	2.00	0.91	80.00%
Marital status	31	1.95	0.88	80.00%	32	2.03	1.00	77.50%
Ambiguity in the role being performed	32	1.93	0.86	82.50%	28	2.18	1.26	75.00%
Job overload	33	1.93	0.89	80.00%	41	1.93	0.89	80.00%
Conflicts of responsibilities	34	1.93	1.00	80.00%	38	1.98	1.12	80.00%
Monotonous work	35	1.90	0.81	82.50%	39	1.95	0.90	82.50%
Information and control	36	1.90	0.87	82.50%	36	1.98	1.00	80.00%
Pressure of time-limits	37	1.90	0.87	82.50%	37	1.98	1.00	80.00%
Lack of autonomy in decision-making	38	1.88	0.79	80.00%	34	2.00	1.04	77.50%
Poor relationships with colleagues and superiors	39	1.85	0.74	80.00%	44	1.90	0.74	77.50%
Personality type A	40	1.85	0.77	82.50%	35	1.98	0.95	77.50%
Poor leadership	41	1.83	0.71	82.50%	40	1.95	0.90	77.50%
Negative results, treatment and uncertainty in patients	42	1.83	0.75	80.00%	51	1.83	0.75	80.00%
No free time	43	1.83	0.81	80.00%	47	1.85	0.86	80.00%
Organisational environment	44	1.83	0.87	85.00%	52	1.83	0.87	85.00%
Unclear requirements	45	1.83	0.87	80.00%	50	1.85	0.92	80.00%
Lack of personal control over the environment and daily decisions	46	1.83	0.98	80.00%	54	1.83	0.98	80.00%
Time given to carry out tasks	47	1.80	0.69	85.00%	30	2.08	1.07	72.50%
Afraid to make mistakes and be reported	48	1.80	0.76	85.00%	46	1.88	0.91	82.50%
Poor communication and unclear job expectations	49	1.80	0.76	80.00%	45	1.90	0.90	75.00%
Insufficient financial compensation for the work performed	50	1.80	0.79	82.50%	42	1.93	1.00	77.50%
Doctor and nurse conflicts	51	1.80	0.79	82.50%	56	1.80	0.79	82.50%
High physical demands	52	1.80	0.97	82.50%	43	1.90	1.17	80.00%
Mental overload due to the continuous concentration that certain patients require	53	1.78	0.73	87.50%	49	1.85	0.89	85.00%
Night shifts	54	1.78	0.73	82.50%	48	1.85	0.86	80.00%
Few opportunities for promotion and growth	55	1.75	0.81	87.50%	58	1.75	0.81	87.50%
Inadequate preparation for certain services	56	1.73	0.88	82.50%	55	1.83	1.06	80.00%
Does not perform leisure or recreation activities	57	1.70	0.76	87.50%	57	1.78	0.92	85.00%
Does not sleep sufficient hours	58	1.68	0.62	92.50%	53	1.83	0.98	90.00%
Need for social support	59	1.63	0.93	85.00%	59	1.63	0.93	85.00%
Work complexity	60	1.50	0.55	97.50%	60	1.50	0.55	97.50%

## Discussion

### Main findings

The interest of researchers and practitioners in burnout has increased significantly over the first 15 years of the 21st century. This is most likely due to the negative consequences that burnout has both for the individual and the organisation. Different authors have proposed different ways of categorising factors antecedent to burnout [48]. However, researchers seem to agree that factors related to work are key in the development of burnout. The work environment is the most studied antecedent to burnout [49]. This may be why 24 (71%) of the 34 factors classified by experts as ‘well studied’ are related to the work environment. The remaining factors classified as ‘well studied’ are socio-demographic and associated with lifestyle, two of these remaining factors are related to personality (type A personality and the need for social support).

According to Furingsten, Sjogren, Forsner [50], burnout is not only caused by work-related factors. Other factors such as lifestyle and personality traits also contribute to the occurrence of the syndrome. They do not occur in isolation and their association with burnout may be reliant on the presence or absence of another factor. Added to this, some of these factors may have a direct influence on burnout, whereas others may have an indirect one, by means of mediating or moderating relations. In regard to lifestyle, there seems to be a consensus among researchers that continuously witnessing pain and suffering requires great mental balance and a full life outside of work. Some studies have researched this relationship [51]. Furthermore, as indicated by numerous authors, factors related to the individual's personality should be taken into consideration when explaining burnout [52]. The most studied personality trait related to burnout is probably the type A personality. Subjects with type A personality tend to perceive the environment as contrary to their goals and threatening to their self-esteem. Type-A personality is a behavioural pattern that is prone to action, to domination with a strong inclination to compete in addition to expressing strong proclivity to hostile reactions [53]. According to experts, another well-studied feature of personality is the need for social support. Although there is no single definition, social support refers to the presence or absence of psychological support that can be found in significant others [54]. The person needs to believe that they are cared about and loved, esteemed and valued, and that they belong to a communication network where there are mutual obligations. It references the degree to which basic social needs are met through interaction with others [55].

The reason for the consensus on the classification of socio-demographic factors may be because much of the research into the syndrome analyses whether the degree of burnout in nurses differs according to sex, age, marital status, years of professional experience or level of education. This occurs regardless of whether burnout factors relate to nurses' working environment, personality factors or lifestyle. However, the findings are not consistent. Moreover, there are confounding variables (e.g., age and work experience, sex and type of occupation) that make the interpretation of any demographic results more difficult [56].

Ten of the factors (58.8%) classified as ‘studied little’ by the experts are internal factors relating to the individual (internal- individual-related-factors). The remaining seven factors (41.2%) are related to the organisation and work environment. Although the factors related to working conditions play are large role in the development of burnout, the way work stressors are perceived and interpreted contributes to burnout. Kanter’s theory [57] argues that people who perceive that their work environment provides them with access to information, support and the necessary resources are empowered, contributing to protecting individuals from suffering burnout [58]. Whereas, in Spreitzer’s theory [59], burnout is not so much determined by the characteristics of the working environment, but rather by the psychological interpretation that the person gives to such an environment (psychological empowerment). Therefore it is not surprising that researchers have described and studied the influence of factors related to personality traits when exploring burnout [60]. Laschinger, Finegan, Shamian, and Wilk [61], using a longitudinal design, showed that structural empowerment has a direct effect on psychological empowerment, and it has a direct effect on burnout. Therefore, structural empowerment has an indirect effect on burnout. Factors related to personality and socio-cultural values [42–43] act as modulators between the work environment experienced by the nurse and their consequent physical/psychological correlates. Their role is facilitator or inhibitor of the action stressors being played out on the individual. That is, the degree of stress perceived by the individual increases or decreases according to their certain personality traits and/or their personal values, consequently affecting the origin and development of the syndrome.

There is some agreement among researchers that the professionals who are most vulnerable to suffering burnout are empathetic, sensitive, humane, dedicated to their profession, idealistic, altruistic, self-denying, obsessive, enthusiastic and susceptible to identifying with the others [62]. These characteristics, which are inherent to people who feel attracted to the nursing profession, may lead them to experience high degrees of compassion fatigue or compassion satisfaction. Nurses who experience a high degree of compassion satisfaction in their work perceive a more positive working environment, which contributes to the reduction of burnout. On the contrary, those experiencing a high degree of compassion fatigue perceive a more negative working environment thus experimenting a higher degree of burnout [63]. Moreover, recent research has shown that optimism, hardy personality or emotional competence are factors that protect the individual against burnout. Positive Psychology claims that the negative effects of stressors related to work environment and daily life could be avoided, at least partially, if nurses were aware of their personal strengths. This means emphasising the positive aspects of individuals (optimism, hardy personality, emotional competence, etc.) in order for these positive emotions to act as a shock absorber in the event of adversity [64]. However, the issue about how the specific features of a personality affect the perception of burnout remains unclear [65]. Personality variables, in addition to having a direct effect on burnout, also play a moderating role [27]. This may explain why, in the opinion of experts, the study of these factors that promote the syndrome have been understudied.

Organisational factors classified as ‘studied little’ have a common denominator: a lack of clarity in the information provided to nurses. This lack of clarity refers to both strategic pillars (mission and vision) and tactics (processes and procedures). In the long-term, this lack of clarity leads to a feeling of exhaustion and fatigue as individuals are obliged to participate in procedures and processes whose ultimate purpose is unknown [66].

Of the nine factors classified as ‘studied very little’—of which eight were included by the experts in the first e-Delphi round—seven (77.8%) are related to the organisation and work environment, and two to events in their daily lives. This result shows that despite the large number of publications devoted to stressors related to the work environment, there are still others requiring attention. A justification for the discrepancy between experts' opinions and the attention given by researchers to some of these variables may be due to the absence of standardised records revealing the time spent by the nurse doing tasks which are not recognised (invisible care), bureaucratic tasks and unwanted distractions from work due to continuous interruptions. The non-equitable workload, the mental concentration and alertness and the continuous adaptation to new technological procedures are situational factors very much related with the unit in which a nurse works (e.g. critical care vs traumatology). The first two are potentially difficult to modify and may be contemplated as non-adequate objectives for intervention. This may be the reason why they have been previously under-investigated. As regards technological advancements, some authors have shown that they could facilitate nursing care, but also that they could sometimes be an obstacle to offer quality care [67]. And yet, the need to adapt to the continuous technological changes may generate a perception of autonomy loss and provoke stress [68]. This situation might be controlled by improving the initial and continuous education in the use of new technologies which would efficiently respond to the need of attention that patients require.

The feminine stereotype, the economic difficulties and the illness suffered by the subjects or their children in the past 12 months are the other 3 factors which, in the opinion of experts, have been under-investigated. Concerning the first of those factors (the feminine stereotype) the Social Dominance Theory, which roots in the idea that all human societies tend to structure in hierarchical systems which have one keeping the hegemony over the rest [69], could explain why this is a potentially difficult factor to modify and could be a non-suitable target for intervention. As Tausch and Hewstone claim [70], the modification of stereotypes is very difficult as it is negatively related with the wish to maintain the social hierarchies. The other two factors, that is, the economic difficulties and the illness suffered by the subjects or their children in the past 12 months, are related to the life of the individual and evince the work-family conflict. There are barely no works having examined the nature of the relation between the work-family conflict in nursing and burnout [71]. However, the limited empirical data shows rather high proportions of work-family conflict among nurses [72]. Hospital management could contribute to diminish the negative effects on the nurses' health by developing policies and practices which would facilitate the successful combination of work with private life for employees [71].

The two top factors in the classification that have not been sufficiently studied by researchers is a lack of recognition of part of the tasks that nurses perform (invisible care) and feminine stereotype. The lack of recognition for much of the work that nurses perform and a work environment that is increasingly difficult to cope with, result in a progressive loss of idealism and energy, reasons that initially led nurses to choose that profession [2].

The nursing profession is under-recognised both socially and professionally [73] and nurses' wages as well as working conditions are often inadequate [74]. Little is known of the competences and degree of autonomy and independence of these professionals [75]. There are even problems with the definition of what a nurse is in all countries of the world [76]. This comes from the commonly held opinion that nursing is inferior to medicine and that the former works in the shadow of the latter. Most people are unaware of the competences inherent in nursing and only trust this profession in functions or activities that have been assigned to it throughout its historical background [77]. Nurses carry out many functions that are scarcely different from those performed by other groups involved in patient care; which can lead one to think that nurses are capable of everything and continuously change the service they work in. In each of the countries of the experts consulted, there are nursing associations and unions. Unfortunately, due to the fact that these institutions have focused on the defense of individual interests, most nurses have not been taught the need to act in concert with others so that society knows the work and functions performed by nurses in order for their performance to be acknowledged by the public [74]. Moreover, current reimbursement systems are in favour for technical and invasive interventions rather than for caring aspects. Thus, also Hospital Administration is focused more on services provided by doctors than on nurses [78].

Nursing staff have been identified publicly as a profession made up of women. In Canada and the United States, only 5% of nurses are males [79, 80]. In European countries, between 5% and 10% of nurses are men [81]. A significant number of previous studies have tried to establish the relationship between gender and burnout in the nursing profession. Although some have found that no significant relationship exists between the gender of nurses and burnout, the majority of them corroborate this relationship and show a higher prevalence of burnout in women compared with men [82]. However, it does not explain to what extent the gender stereotypes, so prevalent in the nursing profession, are a cause of burnout [83]. This stereotype still conditions the functions of this profession which has always been overshadowed by the doctor. On the other hand, this stereotype has contributed to the deterioration of the profession’s image, both internally and externally, and is hampering the relationship, in conditions of equality, of the nurses with other health professionals [84].

Among nurses generally, there is consensus that nursing is a profession that has difficulty in making itself visible and recognisable [85]. One of the reasons for this situation is that very often part of the care provided (emotional and psychological care) is “invisible”. Nurses constantly carry out “invisible” actions that are specific to their profession that are neither instructed nor recorded, actions that include counselling, consolation and showing concern for someone. Invisible care goes unnoticed and languishes without the recognition it deserves. And yet, according to Lanquetin [86], it represents up to 72% of the working time of the nursing staff. The difficulty to gain visibility and make visible the emotional and psychological care they provide, which results in a major gap between the work that is done and the work that is recognised, is gradually deteriorating the quality of the service provided by the nurse and leads to the onset of burnout syndrome symptoms. This gap can be interpreted by nurses as a violation of so-called psychological contract [87]. Consequently, the nurse experiences a lack of reciprocity that may come from either the patients or the organisation. The perception that there is a decompensation between the efforts and the rewards obtained in return is essential to the development of burnout [27, 19].

According to Fairclough [88], the invisible care that accompanies medical actions is undervalued, which in turn contributes to reducing the professional fulfilment of the nursing staff and exacerbates emotional exhaustion and depersonalisation. Another aspect that contributes to the occurrence of burnout is the feeling suffered by nurses of permanent inequality in the eyes of society and patients due to not knowing or not having transmitted reliably the competences that correspond to them [1].

Our finding possibly summarises experts’ personal experiences and theoretical as well as research (current or future) perspectives. It also reflects the influence cultural, economic and political factors in their countries of origin have on the experts [89]. Future research should consider these factors. The majority of these factors would require a careful analysis to be measurable. Under-investigated factors should not be the focus of future research at the expense of currently established predictors, correlates or antecedents of burnout. It would be much more interesting to look at the interaction between the under-investigated factors and those already known. Moreover, it would be interesting to make use of integrating models, which would include individual variables much as organizational ones and would furthermore consider the emotional overload resulting from patient contacts, the lack of reciprocity between what nurses give and take in exchange, or the emotional contagion [10, 19]. Besides, it would be advisable that these models were tested using longitudinal designs, which would allow to establish the directionality of relations amongst variables. An extensive theoretical/conceptual unpacking could help to: a) improve the operational assessment of the construct; b) develop other perspectives that enhance understanding of the syndrome in the field of nursing [12] and; c) enable the development of management strategies to improve nurses’ experiences.

The high and rapid consensus among experts could indicate that the attention that researches give to the factors influencing burnout does not differ significantly regardless of the cultural, economic and/or political differences in their countries of origin. This does not mean, however, that the same factor, depending on the context, may influence the development process of the syndrome in different ways. Future research should identify how context modulates the importance of these factors in the development of burnout.

### Strengths and limitations

Using the e-Delphi technique has allowed us to access information of a subjective nature, which is relevant for classifying the factors affecting burnout in nurses according to the level of attention it is given by researchers. This study involved 40 experts from 6 different countries, with different socioeconomic contexts. Their previous professional experience (more than 15 years' experience in the profession) and their research experience suggest that our panel is sufficiently broad, experienced and capable of making accurate judgments. Moreover, the high level of consensus for each of the 60 factors considered in the study, as well as the high value given by the total mean qualitative group stability support the credibility of the classification made by the experts.

As with every research, this study has some limitations that are worth noting. The first of these is that only studies published in English were included. This may have contributed to leaving out some factors that could be relevant in explaining burnout. Secondly, the sample is of convenience, composed of experts belonging to countries of the European Union. These countries have a more individualistic culture than that of developing countries, which are more collectivist societies according to the terminology of Triandis et al., [40]. This may affect the importance that each culture gives to the factors involved in the onset of the syndrome and their interpretation, limiting the generalization of the results to developing countries. Added to this, despite the similarity of factors that have been related to nurses' burnout by researchers in Europe, the United States [63] or Australia [90], it may occur that the distinct cultural differences, social politics or economic conditions among them may affect the attention given to the specific ones influencing burnout in nurses.

## Conclusion

This research study has shown that there are factors which are important to explaining burnout in nurses and yet have received little attention from researchers. This shows that for a better understanding of the phenomenon further research, both quantitative and qualitative, is needed. These investigations should not forget the analysis of the relationship between burnout and less studied factors
